# Novel Perspectives on the Characterization of Species-Dependent Optical Signatures of Bacterial Colonies by Digital Holography

**DOI:** 10.1371/journal.pone.0150449

**Published:** 2016-03-04

**Authors:** Igor Buzalewicz, Małgorzata Kujawińska, Wojciech Krauze, Halina Podbielska

**Affiliations:** 1 Faculty of Fundamental Problems of Technology, Department of Biomedical Engineering, Bio-Optics Group, Wrocław University of Technology, Wrocław, Poland; 2 Department of Mechatronics, Institute of Micromechanics and Photonics, Warsaw University of Technology, Warsaw, Poland; Institute for Sustainable Plant Protection, C.N.R., ITALY

## Abstract

The use of light diffraction for the microbiological diagnosis of bacterial colonies was a significant breakthrough with widespread implications for the food industry and clinical practice. We previously confirmed that optical sensors for bacterial colony light diffraction can be used for bacterial identification. This paper is focused on the novel perspectives of this method based on digital in-line holography (DIH), which is able to reconstruct the amplitude and phase properties of examined objects, as well as the amplitude and phase patterns of the optical field scattered/diffracted by the bacterial colony in any chosen observation plane behind the object from single digital hologram. Analysis of the amplitude and phase patterns inside a colony revealed its unique optical properties, which are associated with the internal structure and geometry of the bacterial colony. Moreover, on a computational level, it is possible to select the desired scattered/diffracted pattern within the entire observation volume that exhibits the largest amount of unique, differentiating bacterial features. These properties distinguish this method from the already proposed sensing techniques based on light diffraction/scattering of bacterial colonies. The reconstructed diffraction patterns have a similar spatial distribution as the recorded Fresnel patterns, previously applied for bacterial identification with over 98% accuracy, but they are characterized by both intensity and phase distributions. Our results using digital holography provide new optical discriminators of bacterial species revealed in one single step in form of new optical signatures of bacterial colonies: digital holograms, reconstructed amplitude and phase patterns, as well as diffraction patterns from all observation space, which exhibit species-dependent features. To the best of our knowledge, this is the first report on bacterial colony analysis via digital holography and our study represents an innovative approach to the subject.

## Introduction

A variety of biosensors are routinely used for bacterial detection and identification [1–4]. Optical methods do not require contact and allow for non-destructive inspection, which is why they are intensively studied for the development of novel inexpensive and rapid detection techniques for bacterial pathogens in food, water, or clinical samples. Over the past few years, significant progress has been made using light scattering on bacterial colonies [5–10].

We previously demonstrated that colonies from different bacterial species and strains generate specific diffraction signatures that can be used for microbiological diagnosis, as both the amplitude and phase spatial light are species- and strain-specific [7,11]. The experiments performed using our system with converging spherical wave illumination have shown that the Fresnel diffraction patterns of colonies were unique and allowed for species discrimination [12–16]. The proposed statistical analysis algorithm for Fresnel diffraction pattern analysis enables identification with nearly 99% accuracy [17]. Moreover, experiments performed by other researchers on four different strains of *Escherichia coli*, also demonstrated that it is possible to identify bacterial strains with a 82% recognition rate [9]. Recently, experiments on bacterial cultures causing infections of the urinary tract have shown that the optical system configuration can be applied in a clinical context, as was originally proposed by our group [18]. Further enhancement of the bacterial identification accuracy on the level of strain discrimination is possible by means of optical diffraction; however, an extended study of the interaction between bacterial colonies and light is necessary.

The main goal of this paper is to extend optical examinations by determining the amplitude and phase properties of bacterial colonies using a Point-Source Digital In-Line Holography (PSDIH) sensor. Recently, we demonstrated the suitability of digital holography for bacteria characterization [20]. This technique may be also useful for the examination of other biological samples [21–24]. Optical holography is a method that allows registration and reconstruction of both the amplitude and the phase of an optical field. Thus, it is possible not only to record the amplitude- and phase-light-modulating properties of an object, but also to obtain information on the amplitude and phase of the transformed optical field. Significant advances in optoelectronics and computer sciences have led to the digitalization of holography [19,20]. The presently used digital holographic microscopes (DHM) capture a hologram with an electronic device, such as a CMOS or CCD camera, and then, the recorded hologram is reconstructed numerically using diffraction calculations. This microscopic technique enables lensless imaging of an object and retrieval of information about the amplitude and phase of the scattered optical field. The simplest configuration is represented by digital in-line holographic microscopy (DIHM). This concept is based on a technique originally proposed by Gabor under the assumption of light scattering on an in-line illuminated object [21–23]. In this case, the interference occurs when the amplitude of the scattered wave is small compared with the amplitude of the non-scattered reference wave.

**Fig 1:**
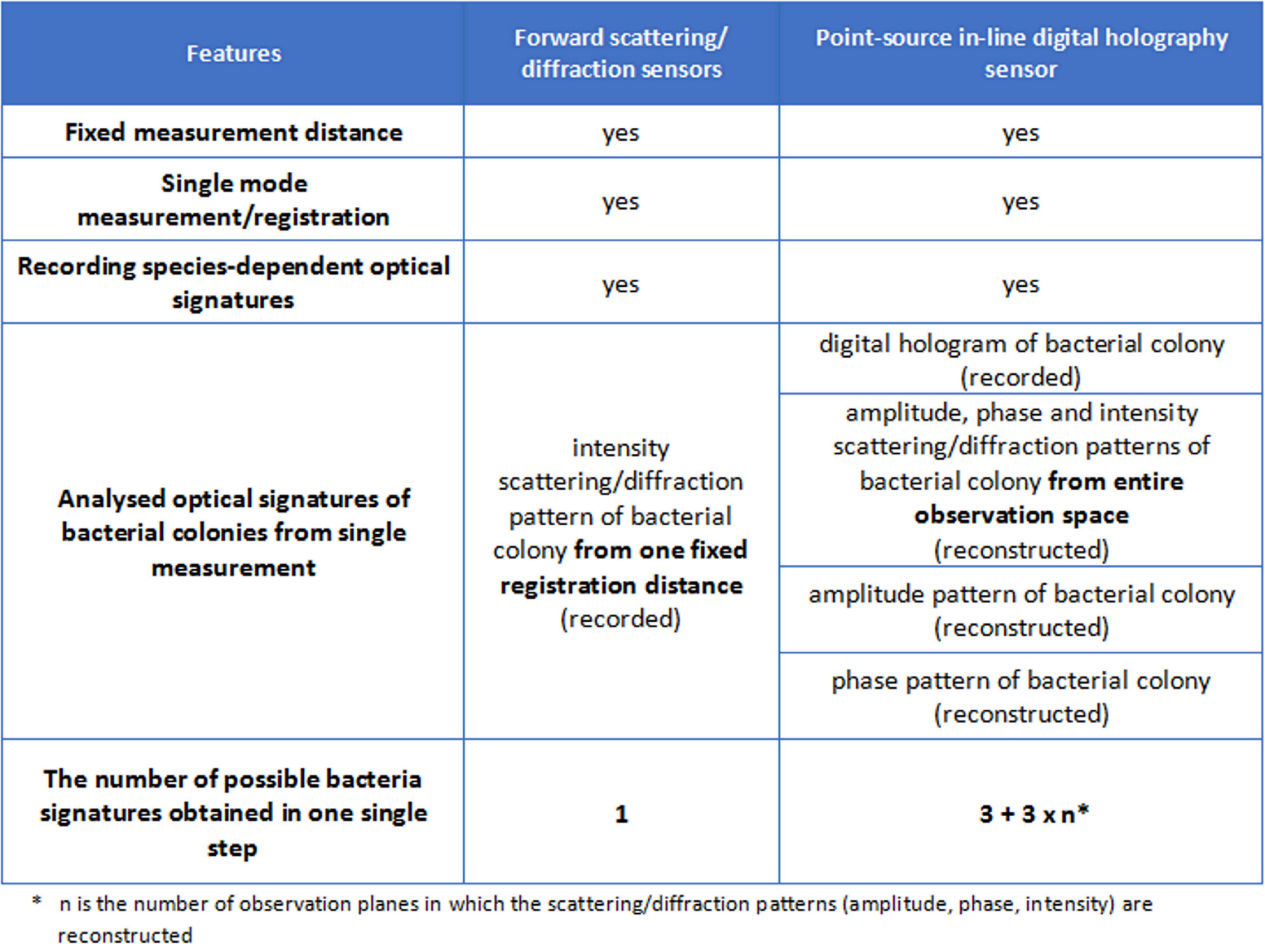
The comparison of already proposed forward scattering / diffraction sensors and novel digital holography sensor.

In our recent work, the PSDIH sensor was applied to study colony-light interaction. This technique allows to generate intensity images of examined objects with sub-micron resolution [22–26]. However, a hologram reconstruction also gives access to the phase information of an object and allows the creation of images that represent the spatial variation of phase shifts of the optical wave fronts modified by an object under study. The main advantage of the DHM is the quantitative determination of the phase shifts, which enables the quantitative analysis of the optical path through the object. This cannot be achieved with a standard optical phase microscope. Moreover, the PSDIH sensor allows reconstruction of the diffracted optical field in all desired observation planes behind an object, based on the recorded single digital hologram and the proper model of diffracted beam propagation. Existing techniques are based on light scattering/diffraction in one selected direction and at a fixed distance from the colony, although the spatial distribution of scattering/diffraction patterns is significantly affected by the observation distance [16]. During the single measurement only one diffraction/scattering pattern can be recorded. Contrary to bacterial identification by forward light-scattering sensors based on diffraction intensity patterns recorded in selected observation planes, DHM techniques enable the analysis of both amplitude/intensity and phase patterns of scattered/diffracted optical fields in the entire observation space. To the best of our knowledge, this is the first attempt to use the PSDIH based sensor for bacterial colony investigation. Colonies of two different bacterial species, *Escherichia coli* and *Staphylococcus intermedius*, are analyzed with both PSDIHM and classical optical microscopy in order to characterize their amplitude and phase properties. Furthermore, the process of hologram reconstruction is applied to evaluate the spatial distribution of the optical field diffracted by a colony in various observation planes behind the complex object. In comparison to the already proposed scattering/diffraction method for bacteria identification, here it will be demonstrated that digital holography enables obtaining new optical signatures of bacterial colonies: digital holograms, reconstructed amplitude and phase patterns, and their diffraction patterns from the entire observation space. These unique features can be treated as novel bacterial species optical discriminators. The aim of the study is to demonstrate the new perspectives in the characterization of species-dependent optical signatures. Contrary to bacterial identification by forward light-scattering sensors, the DHM enables reconstruction of multiple optical signatures of bacterial colonies from one single digital hologram (see Fig 1). This approach may result in the development of new diffraction/scattering methods for bacterial characterization.

## Materials and Methods

### The point source digital in-line holographic sensor

**Fig 2:**
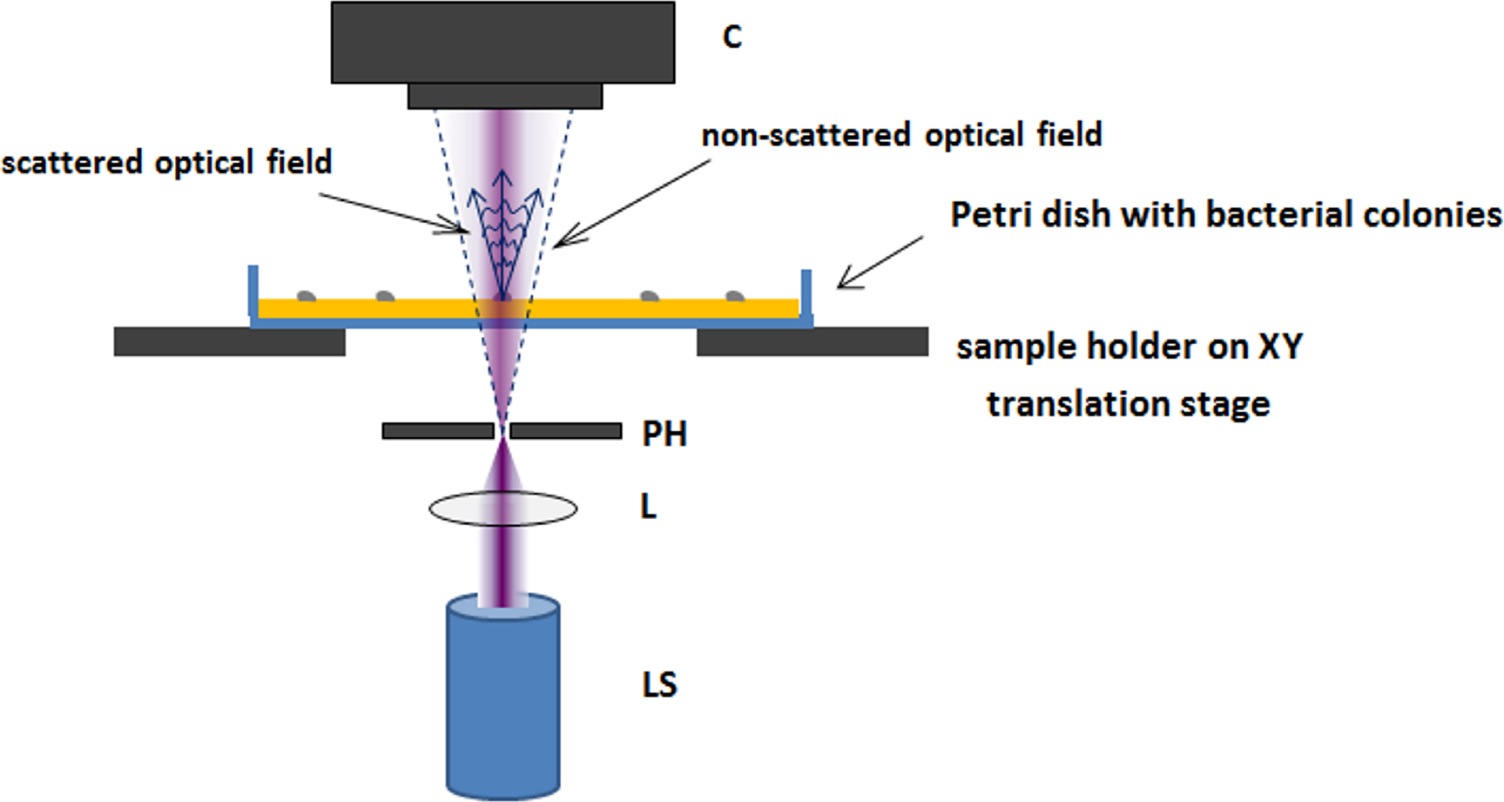
The schematic set-up of the PSDIHS configuration. Light generated by a coherent light source (LS) with a wavelength of 405 nm and output power of 25 mW is focused onto a 0.5-μm-diameter pinhole (PH), which acts as the point source emitting the divergent spherical wave. The wave illuminates an examined object, bacterial colonies grown on nutrient medium in a Petri dish on a sample holder with an X-Y translation stage, and forms magnified diffraction pattern in the recording plane (C) of the CMOS camera with the sensing area: 2048 × 2048 pixels (pixel size: 6 × 6 μm), mounted on a Z translation stage.

The schematic configuration and description of the point-source digital in-line holographic sensor (PSDIHS) used in our study is depicted in Fig 2. Holograms were recorded at a distance of 25 mm from the surface of the nutrient medium.

### The hologram numerical reconstruction algorithm

We adopted the numerical reconstruction of holograms based on the Helmholtz-Kirchhoff transform, originally proposed in [23]:

$$\text{K}\left(\bar{\text{r}}\right)=\int_{S}\text{I}\left(\bar{\xi }\right)\;\text{exp}\left\{2\pi \text{i}\bar{\xi }\cdot \bar{\text{r}}/\lambda \xi \right\}{\text{d}}^{2}\xi$$ (1)

where the integration extends over the two-dimensional surface of the camera detector (perpendicular to the optical axis) at distance L from the pinhole, with coordinates *ξ* = (*X*, *Y*, *L*), λ is the light wavelength, and $\text{I}\left(\bar{\xi }\right)$ is the intensity pattern of the hologram.

In previous applications [23–26], $\text{I}\left(\bar{\xi }\right)$ represented the contrast image (hologram) in the camera plane, obtained by subtracting the holograms with and without the object presence, or by applying the high-pass filter to the hologram. In the current study, the initially recorded hologram pattern was used as the input signal for the numerical reconstruction. This approach will be discussed further in the next sections. The function $\text{K}\left(\bar{\text{r}}\right)$ is the complex amplitude of a wave anywhere in space, in particular within the volume of the object, thus rendering its three-dimensional structure. Generally, $\text{K}\left(\bar{\text{r}}\right)$ can be determined in an arbitrarily chosen plane in the space behind the object and it can be used for the analysis of the spatial intensity distribution of the complex amplitude of the light diffracted by the object. A reconstruction plane that contains the image of an analyzed object’s cross-section is equivalent to the in-focus plane in conventional optical microscopy. The term $\text{K}\left(\bar{\text{r}}\right)$ is a complex function and the intensity KK* represents the object. The phase image of an object is obtained by determining the phase angle Ψ(r), as described by the following expression:

$$\psi \left(r\right)\;=\;arctan\frac{Im\;K\left(r\right)}{Re\;K\left(r\right)}$$ (2)

### Preparation of the bacterial colony samples

The samples were prepared in the microbiology laboratory of the Department of Environmental Engineering at the Warsaw University of Technology. Bacterial suspensions of *Escherichia coli* (PCM 0119) and *Staphylococcus intermedius* (PCM 2405) were first incubated for 24 h at 37°C. Respectively, 10^−6^ and 10^−7^ dilutions were seeded on the surface of Columbia agar (Oxoid) solid nutrient medium in Petri dishes to obtain 10–15 colonies per plate, and were incubated at 37°C for 20 h. To avoid variation in light attenuation, the same volume of nutrient medium was used in each case (10 ml).

### Spectroscopic study of light attenuation by nutrient medium and bacterial colonies

A spectroscopic study was performed in the spectral range of 300–800 nm with a resolution of 2 nm. The absorption spectra of bacterial colonies were measured using an AvaSpec 3648 spectrometer (Avantes) with a reflection probe. The absorption spectra of the Columbia nutrient medium (Oxoid) were recorded by the same spectrometer working in the transmission configuration.

### Examination of bacterial colonies by optical transmission and phase contrast microscopy

To explain the correlation between the reconstructed image, recorded by the PSDIHS, and the structure of the examined bacterial colonies, additional examinations were performed using a Nikon Eclipse 2000 optical transmission and phase contrast microscope (Nikon objectives: 4×, 10×). Based on the images, the transmission coefficient of the bacterial colony was determined. Based on wave optics, the complex amplitude U(x′, y′) of the optical field in the image plane in transmission mode can be described by the following expression [27]:

$$U\left(x^{\prime },y^{\prime }\right)\;=\;\frac{1}{\left|M\right|}U_{0}\left(\frac{x^{\prime }}{M},\frac{y^{\prime }}{M}\right)$$ (3)

where ${\text{U}}_{0}\left(\frac{{\text{x}}^{\prime }}{\text{M}},\frac{{\text{y}}^{\prime }}{\text{M}}\right)$ is the rescaled complex amplitude of the optical field in the object plane and M determines the transverse magnification of the microscope optical system, *x*’ = *Mx* and *y*’ = *My* are relationships connecting the coordinates x, y in the object plane with the coordinates x’, y’ in the image plane. Considering the fact that only the intensity of the optical field is recorded in the conventional transmission microscope, the phase modulation is totally lost. The intensity of the optical field in the image plane can be described as follows:

$$I\left(x^{\prime },y^{\prime }\right)={\left|U\left(x^{\prime },y^{\prime }\right)\right|}^{2}≅\frac{1}{{\left|M\right|}^{2}}T\left(\frac{x^{\prime }}{M},\frac{y^{\prime }}{M}\right),$$ (4)

The microscopic image enables obtaining the rescaled two-dimensional transmission coefficient $\text{T}\left(\frac{{\text{x}}^{\prime }}{\text{M}},\frac{{\text{y}}^{\prime }}{\text{M}}\right)$. Therefore, it is possible to determine the transmission coefficient T(x’, y’) of the bacterial colony, according to the following expression:

$$T\left(x^{\prime },y^{\prime }\right)=T\left(i,j\right)=\frac{I_{ob}\left(i,j\right)}{{\bar{I}}_{p}^{0}}$$ (5)

where T(i, j) is the discrete transmission coefficient for the particular pixel (i, j) of the recorded transmission image of the colony, I_ob_(i, j) is the discrete intensity value of the colony image pixel, and ${\bar{\text{I}}}_{\text{p}}^{0}$ is the average discrete intensity of the pixels of the nutrient medium image outside the region occupied by the colony. T(i, j) is the relative transmission coefficient of the bacterial colony, normalized to the transmission properties of the nutrient medium on which the colonies are grown. The T(i, j) ∈ < 0; 1 > values are dimensionless units. The value 0 describes the situation of total light attenuation for an opaque object, and value 1 describes total transmission for a transparent object.

### The Principal Components Analysis (PCA) of examined optical signatures of bacterial colonies

The special image processing algorithms and features extraction developed in our group, were exploited for the statistical analysis of the recorded optical signatures of bacterial colonies [12,15–17]. The recorded holograms, reconstructed phase and amplitude patterns, as well as the reconstructed diffraction intensity patterns of bacterial colonies were examined. Analyzed patterns exhibit the circular symmetry, therefore they have been processed by the dedicated macro written in ImageJ free software (http://rsb.info.nih.gov/ij) [28] in order to distinguish the center and edges of the patterns. The 5 concentric annulus-shaped zones partitioning the analyzed patterns were used to extract their unique features. Data consist of mean value and standard deviation for each of analyzed zone, so the 10 features of each patterns were examined. For each bacteria species 40 holograms, amplitude and phase images, and reconstructed diffraction intensity patterns were analyzed. The Principal Components Analysis (PCA) as well as a dimensionality reduction of the extracted features and an initial grouping of the data, were performed by means of R free software for statistical computing [29].

## Results and Discussion

### Spectroscopic examination of absorption properties of the bacterial colonies

**Fig 3:**
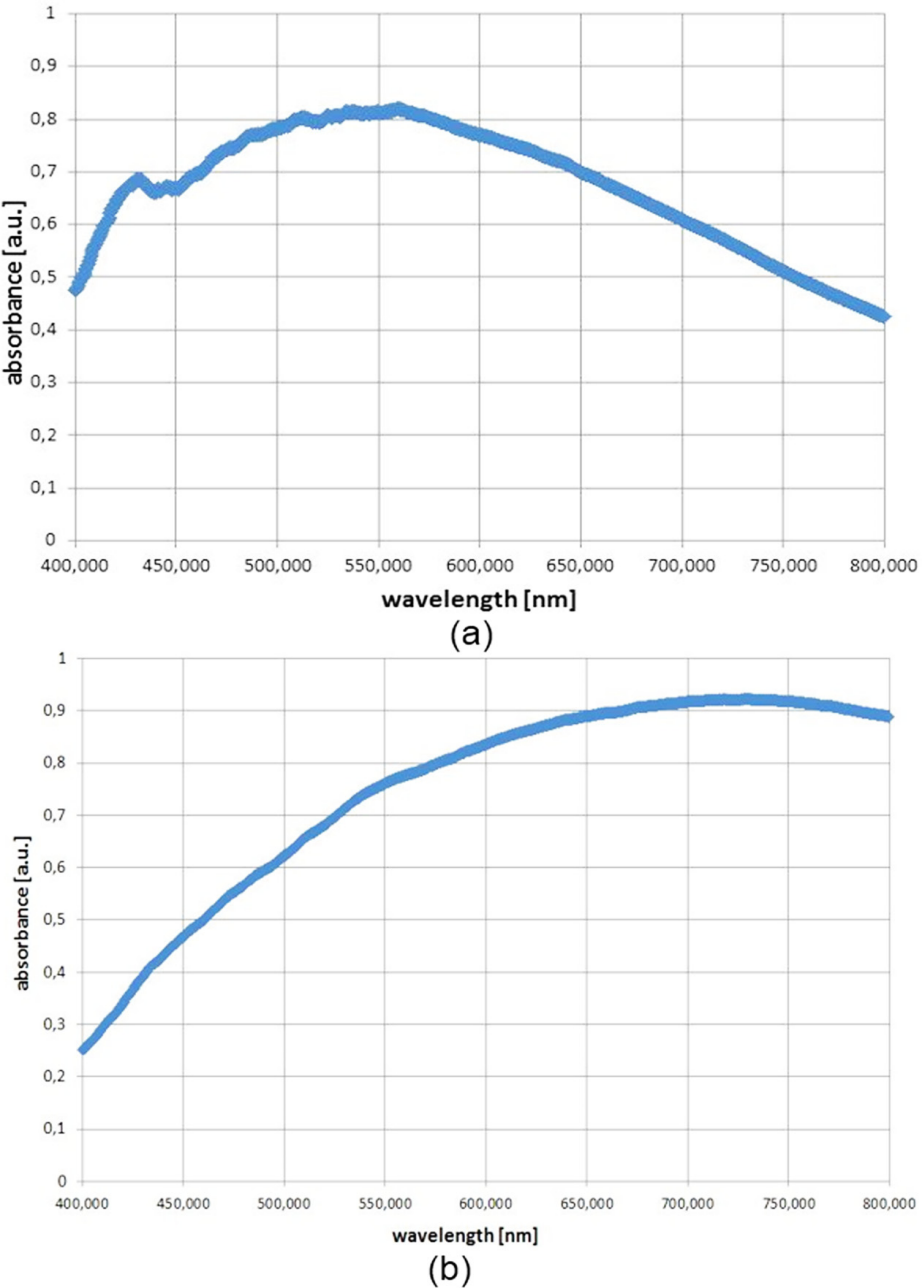
Exemplary reflection absorbance spectra of the bacterial colonies of (a) *S*. *intermedius* and (b) *E*. *coli*.

In PSDIH, it is particularly important to preserve the appropriate transmission properties of the illuminated object to allow the possibility of recording the high-contrast interference patterns of the non-scattered and scattered waves. Therefore, it is necessary to determine the absorption properties of the bacterial colonies (see Fig 3). For the determination of the absorption spectra of the bacterial colonies, the light reflected form the nutrient medium was used as reference.

Previous examinations of the absorption properties of Columbia nutrient medium (Oxoid) demonstrated that this medium has a significant absorbance for the wavelengths from 400 nm to 425 nm [8]. Therefore, a constant volume of the nutrient medium on the Petri dish was used in order to preserve high transmission of the light beam with a wavelength of 405 nm, as used in the PSDIHS.

*Escherichia coli* colonies (see Fig 3B) exhibit absorption in the entire VIS spectral range with the main maximum around 700–750 nm; however, for wavelengths shorter than 450 nm the absorbance decreased to 0.25. Therefore, the constraint of the PSDIHS referring to the object’s transmission can be omitted for light with a wavelength of 405 nm.

For *Staphylococcus intermedius* colonies (see Fig 3A), the maximum absorbance is observed for the wavelengths from 500 to 600 nm and the absorption significantly decreases for longer wavelengths. Moreover, for wavelengths between 400 and 450 nm the absorbance values vary between 0.49 and 0.7. Therefore, the light transmission is more limited for these colonies than for *E*. *coli* colonies. This affects the contrast of the recorded holograms and reconstructed images of colonies.

The light attenuation of *E*. *coli* colonies is not very strong. Moreover, the recorded holograms and reconstructed colonies images were of low contrast. This is associated not only with the absorption properties of the bacterial cells and the extracellular material, but also with the geometry and central thickness of the colony. Limiting the incubation time can improve the quality of the recorded holograms and reconstructed images, since the colony thickness, and consequently the optical path length trough the colony, will be reduced. However, even for the incubation time applied in this study, it was possible to record holograms of the required quality for reconstruction.

### The recorded point-source digital in-line holograms of bacterial colonies

**Fig 4:**
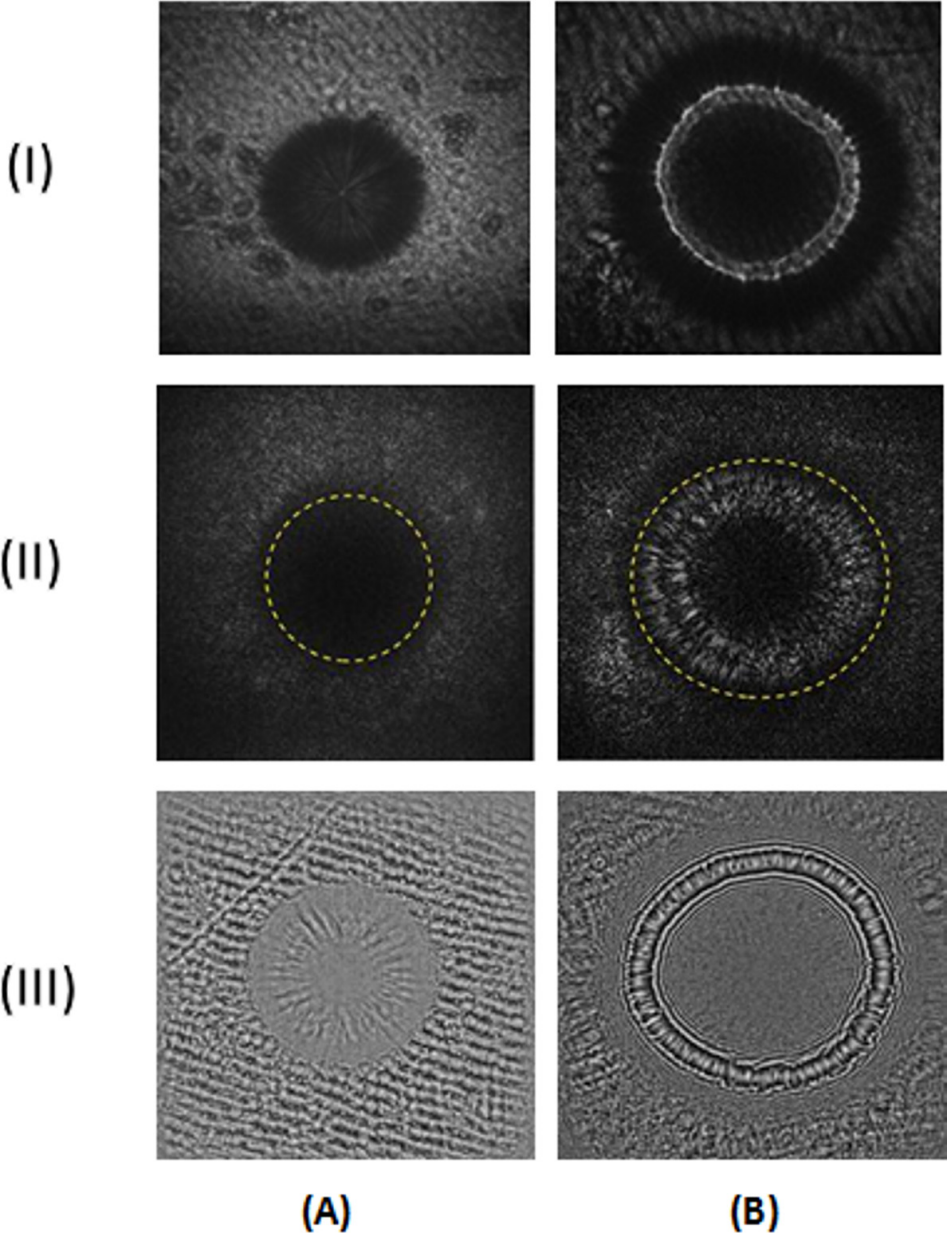
**Representative holographic signatures of *S*. *intermedius* (A) and *E*. *coli* (B) bacteria**. (I) The digital in-line holograms of bacterial colonies, and (II) the reconstruction of the spatial distribution of the optical field’s amplitude inside the space occupied by the colonies (dashed lines indicate the colony contour)). (III) The reconstruction of the spatial distribution of the optical field phase mod (2π) inside the space occupied by colonies.

After obtaining holograms for both bacterial colonies (see Fig 4(I)) it is clear that the contrast between the nutrient medium and the colonies is lower in the case of *S*. *intermedius* than for *E*. *coli*. However, the transmission of *E*. *coli* colonies should be higher, according to the recorded the absorption spectra. This effect can be associated with the colony diameter, which is smaller in case of *S*. *intermedius*, with respect to the constant diameter of the illuminating beam. Bacterial colonies are semi-transparent objects. Therefore, the object beam intensity is expected to be limited in the same way as the reference beam intensity. In spite of this, it is possible to record the holograms of the colonies. However, the hologram background is not as uniform as in for conventional digital in-line holograms of biological objects [22,23,26]. Visual inspection of the recorded holograms revealed that in the region outside of the colonies shadow, an additional interference pattern is observed. The non-uniform dark background of the holograms resulted from the nonhomogeneous structure of the nutrient medium formed during gelation, which introduces an additional phase shift of the transmitted light. Other factors include scratches on the surface of the nutrient medium formed during the bacterial cell seeding procedure. Such imperfections cannot be avoided due to manual execution of laboratory procedures. These factors undoubtedly influence the quality of the reconstructed amplitude and phase patterns, since it is not possible to obtain interference fringes on a uniformly dark background with high contrast, which, in case of other objects, can be achieved by subtracting the background. Of note, reconstruction of the hologram without background subtraction leads to residual interference fringes and artifacts that can be totally avoided if the reconstruction is done from a high contrast hologram [23]. However, in this case, the background subtraction is impossible due to the presence of totally random scattering patterns caused by the scratches on the nutrient medium surface. These problems may be solved only by introducing fully automatic bacteria seeding procedures or by shortening the time of bacterial colony incubation, which may improve light transmission.

### Numerical reconstruction of point-source digital in-line holograms of bacterial colonies

From the recorded holograms of bacterial colonies, it is possible to numerically reconstruct both the amplitude and the phase of the incident optical fields modulated by the analyzed biological objects. Therefore, it is possible to trace back and extract unique scattering patterns of the colonies for each bacterial species/strains in the space between the series of object planes and the hologram recording plane under the assumption of free propagation. This is the main advantage of PSDIH over the already proposed bacterial identification systems based on light scattering/diffraction. The numerical reconstruction can be treated as a procedure inverse to the recording, which enables extraction of the optical fields transformed by bacterial colonies from a 2D recorded hologram on the CMOS chip.

#### The amplitude and phase properties of bacterial colonies examined by PSDIH

Representative PSDIH results are presented in Fig 4(II) and 4(III). The patterns reconstructed at distances of 40 μm for *S*. *intermedius* and of 20 μm for *E*. *coli*. The semi-transparency of the colonies limits the possibility of effective colony detection by PSDIH, however, it enables the analysis of some optical properties of the colonies, which were confirmed by additional examination by classical optical microscopic techniques.

**Fig 5:**
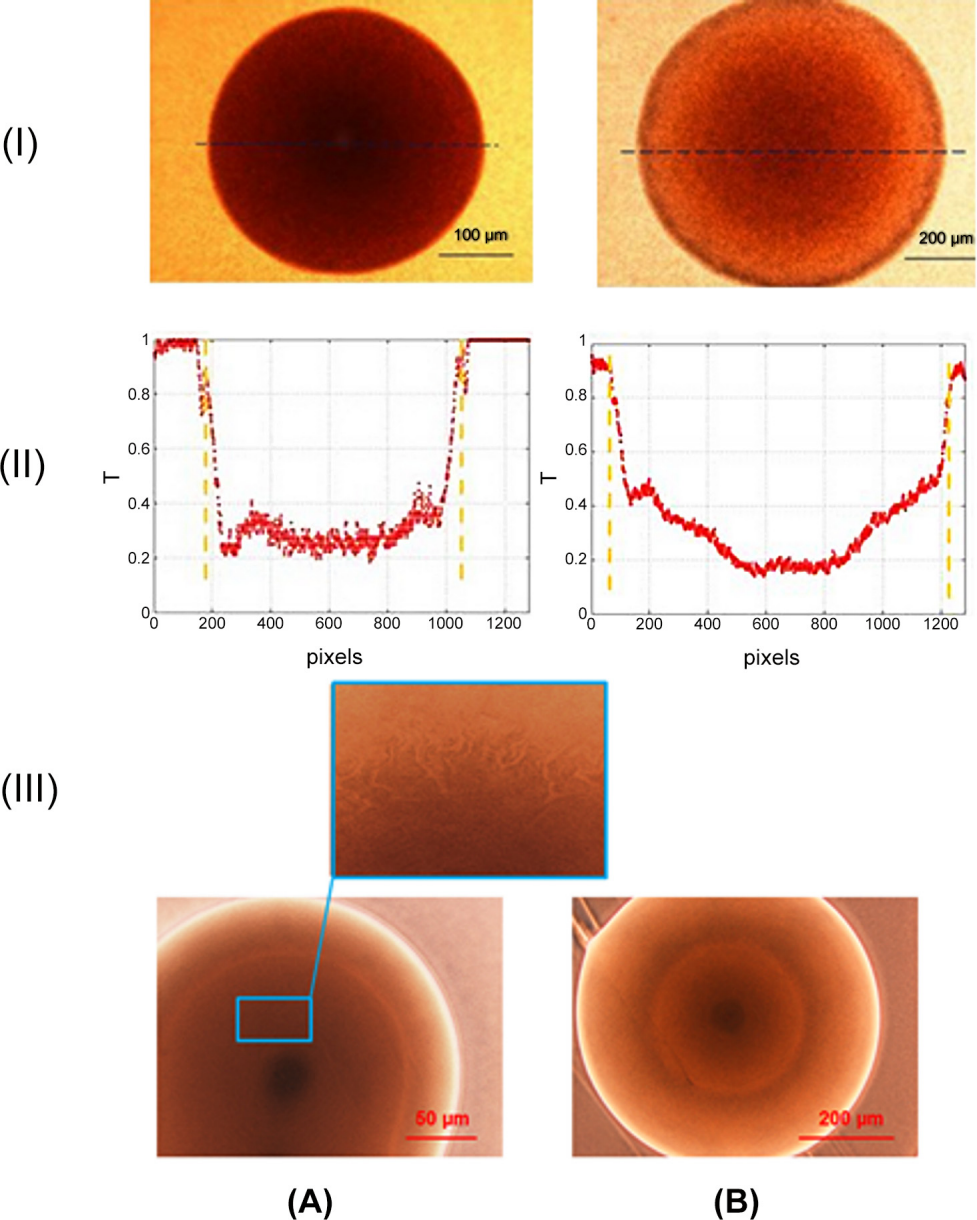
**Representative results of optical microscopic investigation for *S*. *intermedius* (A) and *E*. *coli* (B) bacteria.** (I) Representative transmission microscopy images of bacterial colonies (the dotted lines indicate the cross-section for which the transmission coefficient was evaluated). (II) The transmission coefficient along the cross-section (the dotted lines indicate the bacterial colony edge). (III) Phase contrast microscopy images of (A) *S*. *intermedius* and (B) *E*. *coli* bacterial colonies.

For *S*. *intermedius* colonies, the reconstructed amplitude image only allows for the detection of the shape of the colony, which strictly correlates with high light absorption. For the *E*. *coli* colonies, in contrast, the reconstructed amplitude pattern consisted of a ring-shaped amplitude maximum, which can be associated with the presence of zones inside the colony with different light transmission properties. The transparency of the colony is limited in the central region, where the oldest bacterial cells are located and the colony thickness is the greatest. These observations are confirmed by examination of the colony transmission coefficient by means of optical transmission microscopy (Fig 5(I) and 5(II)). The obtained results indicate that for the *S*. *intermedius* colonies, a significant decrease in the transmission coefficient is observed, from 1.0 on the colony edges to 0.25–0.3 in the colony center. These colonies can be regarded as semi-transparent discs with nearly stepwise changes in transparency. In the case of *E*. *coli* colonies, a decrease in the transmission coefficient is noted, from 1.0 on the colony edges, over 0.40–0.45 in more inner regions, to 0.45–0.2 in the colony center. Thus, in *E*. *coli* colonies, two zones with different transmission properties can be distinguished. These zones can explain the presence of the ring-shaped amplitude maxima in the reconstructed optical field amplitude patterns because of light scattering or diffraction on their edges. In contrast to *S*. *intermedius* colonies, *E*. *coli* colonies can be considered as amplitude filters or semi-transparent discs with gradually changing light transmission regions. This indicates that different transmission properties result from the internal structure of the colony, as well as from the shape of the cells forming each colony. The spheroid shape of *S*. *intermedius* cells may underlie the homogeneous internal structure of the colony, while rod-shaped *E*. *coli* cells, as expected, form colonies with a more heterogeneous structure and with optical properties that vary according to the different spatial orientation of the rod-shaped cells. Therefore, the bacterial cell morphology affects colony structure and the distribution of extracellular material inside the colony. In consequence, the absorption properties of the colonies also affect the light attenuation in different regions of the bacterial colony. Light transmission through the colony depends on the optical path length inside the colony. *E*. *coli* forms colonies with an aspherical profile [20]. Therefore, the highest light attenuation is observed in the central region of such a colony, where the thickness is maximal, and it decreases moving from the center towards the colony edges. *S*. *intermedius* forms colonies with an approximately spherical profile. Therefore, the colony thickness changes less radically than in the case of *E*. *coli* colonies, and the transmission varies less. The light transformation by the colony will be dominated by the knife-edge diffraction on the zones with different transmission properties, indicating that although the conventional imaging of colonies is limited by their transparency, PSDIH can be used to characterize their amplitude properties. As we demonstrated here, colonies of different species exhibit different light transmission regions inside the colony that are responsible for the light-colony interaction.

Analysis of the phase mod (2π) distribution inside the colony shows that each colony has a unique species-dependent phase distribution image (see Fig 4(III)). In case of *S*. *intermedius*, concentric spokes are observed pointing outwards from the center of the colony. These features (spokes) can be associated with the process of colony growth and bacterial cell migration away from the center of the colony, where the oldest cells are located. Local changes in the refractive index (as well as local changes of thickness) can affect the light diffraction of colonies. In case of *E*. *coli*, the phase map has a significantly different structure, indicated by quasi-spherical phase modulation. It contains round fringes with a homogenous structure inside the region of the colony, indicating that the strongest phase modulation occurs on the edges of the zones with different transmission, and can be responsible for the unique spatial distribution of the colony Fresnel patterns (shown in the next sections). This tendency is also observed on phase contrast images of *S*. *intermedius* colonies (see Fig 5(III)), where the changes of the phase image contrast are not as strong as in the case of the *E*. *coli* colonies. The lowest intensity region in the phase contrast images is the center of the colony, where the oldest bacteria are located. The phase contrast image of *S*. *intermedius* colonies exhibits the same unique radial spokes features as those observed in the reconstructed phase patterns. The spokes can be correlated with the migration of the bacterial cells during colony growth. The presence of these features may be caused by the slow dynamic of *S*. *intermedius* colony growth on Columbia medium, as these colonies had a smaller diameter and central thickness than the *E*. *coli* colonies, what enabled the observation of the internal structure of the colony near the nutrient medium surface. Phase images of *E*. *coli* colonies also revealed some darker spots in the central region. The phase contrast images correlated with the spatial distribution of the optical path length inside the colony region and suggest that the profile of *S*. *intermedius* colonies is more spherical than that of *E*. *coli* colonies. Moreover, the most significant phase changes for *E*. *coli* are observed around the central region of the colony, suggesting different light transformation and diffraction in ring-shaped zones of the colony with different transmission properties.

Thus, in terms of classical optics, bacterial colonies can be considered as amplitude and phase light modulators, with different species-dependent amplitude and phase properties, affecting the spatial distribution of the incoming optical field. Moreover, it should be noted that colonies are biological objects that evolve over time during incubation; therefore, their light-modulating properties are also time dependent.

#### Analysis of numerically reconstructed amplitude and phase patterns of the optical field transformed by bacterial colonies

**Fig 6:**
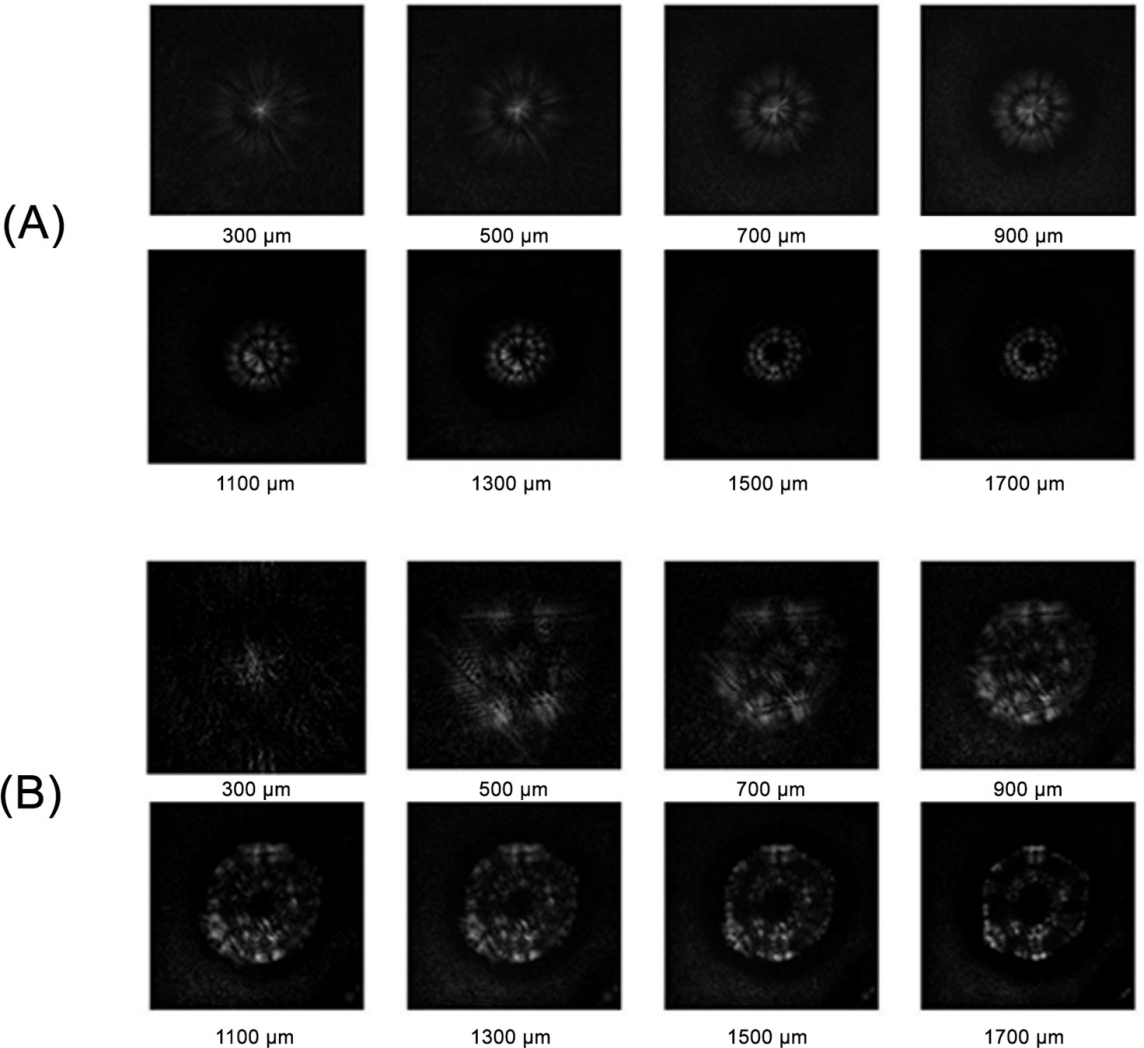
**Representative amplitude patterns of the optical field transformed by *S*. *intermedius* (I) and *E*. *coli* (II) colonies calculated for different observation plane distances**.

A numerical reconstruction was performed according to the algorithm presented in the Materials and Methods section. Representative results are presented in Fig 6. For both bacterial species, it is possible to analyze the propagation of the optical fields transformed by the colonies in arbitrary chosen planes in the observation space behind the object. In each case, the amplitude patterns of the reconstructed diffracted optical field have a different spatial distribution for each location of the observation plane behind the particular colony. These patterns exhibit unique features associated with the morphological and optical properties of the bacterial species under study. Moreover, the convergence of the diffracted optical field, indicated by the size change of the reconstructed amplitude patterns, confirms a previous report [30] on the effect of the geometrical structure of bacterial colonies on their light focusing properties. Therefore, PSDIH reconstruction of the optical field patterns that are scattered/diffracted by the colony can be used for bacterial species identification. In a previously proposed method, the Fresnel diffraction and forward scattering patterns of colonies were recorded for specific locations of the observation plane and used as species signatures. However, for some bacterial species or strains, unique diffraction patterns can only be detected at a specific distance. Therefore, time-consuming measurements of the diffraction/scattering patterns in different locations of the registration plane are required [17]. In contrast, with PSDIH it is possible to record one digital hologram, and numerically reconstruct the amplitude distribution in all desired observation planes. The most adequate amplitude patterns can be chosen to classify different bacterial species without the need of time-consuming recording of diffraction patterns for different observation planes as in the classical optical system. Digital reconstruction enables the analysis of the reconstructed amplitude and intensity patterns that can be regarded as bacteria species classifiers and can be used for bacterial identification by choosing the diffraction patterns exhibiting many species-dependent features. DIH enables the extension of the diffraction pattern features vector and may lead to more complex classification of bacteria species and strains.

#### The correlation of the numerically reconstructed intensity and phase patterns of the optical field transformed by bacterial colonies and their Fresnel diffraction patterns

**Fig 7:**
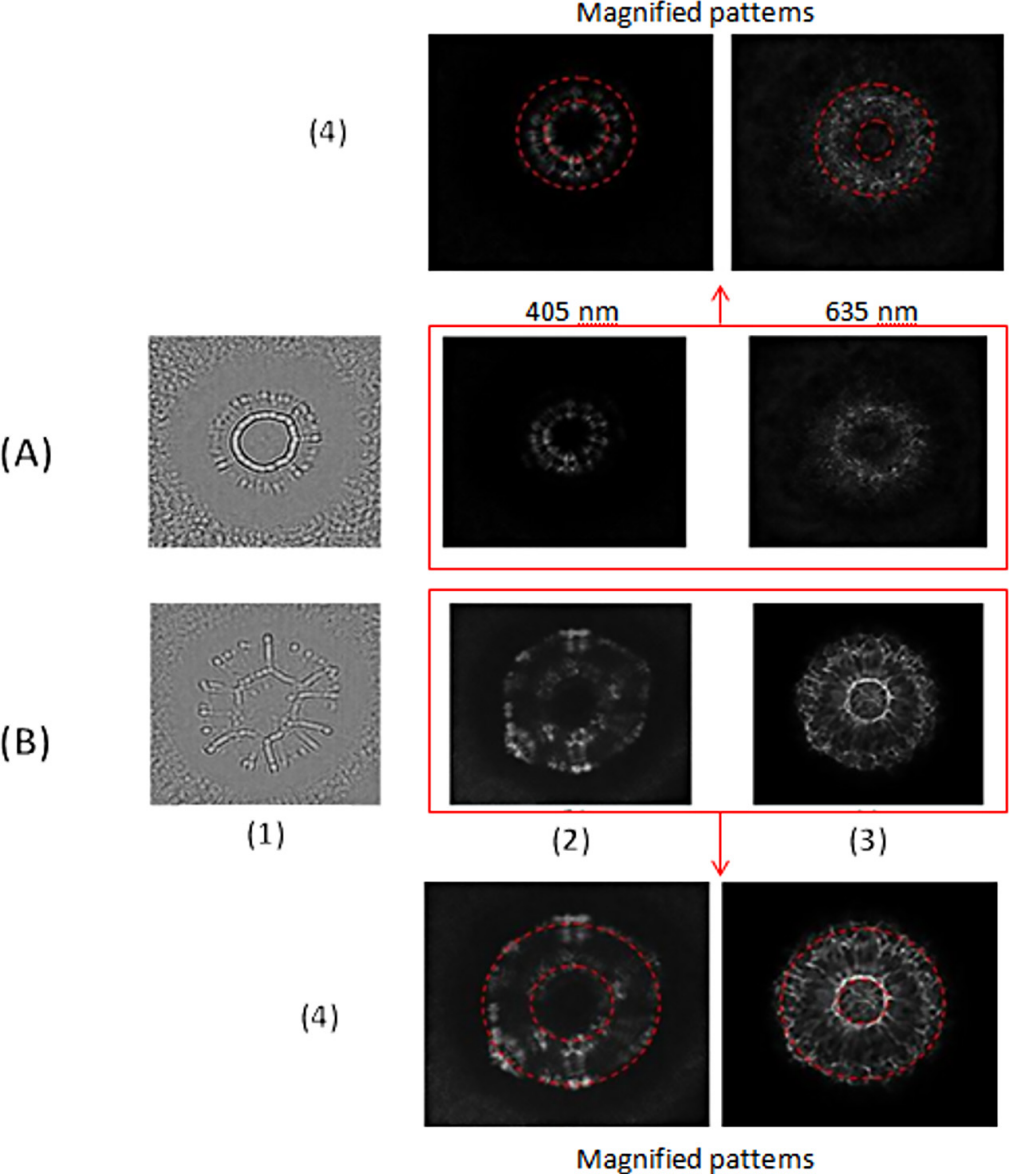
**Representative results for S. intermedius (A) and E. coli (B) bacteria**. (1) Phase and (2) intensity patterns of the optical fields obtained by the reconstruction of the point-source digital in-line hologram at a distance of 2.0 cm from the bacterial colony, and (3) the representative Fresnel diffraction patterns. (4) The demonstration of the wavelength-dependent differences between Fresnel patterns of bacterial colonies reconstructed from digital holograms (at 405 nm) and recorded in an optical system (at 635 nm) (the dashed lines indicate the wavelength-dependent differences of the diffraction rings spacing).

The intensity of the Fresnel patterns of the transformed optical field can be treated as specific optical scattering/diffraction signatures that differentiate bacterial species [16,17]. We determined the correlation between the numerically reconstructed holograms of *E*. *coli* and *S*. *intermedius* colonies using the PSDIH technique, and previously recorded Fresnel patterns (see Fig 7). The reconstructed intensity patterns of the optical fields diffracted by the bacterial colonies exhibit similar unique features as in the case of the recorded Fresnel diffraction patterns. For *S*. *intermedius*, the central low-intensity zone in the diffraction pattern was observed in both the numerically reconstructed and the experimentally recorded intensity pattern, as expected based on the low light transmission in this region of the colony. Therefore, the light transformation by this type of colony is generally only affected by the light diffraction at the edges of the colony, since the phase map of the space region occupied by the colony indicates high homogeneity of its internal structure. The phase modulation of the incoming optical field for this colony depends mainly on the colony profile leading increased convergence of the diffracted field.

For *E*. *coli* colonies, the light diffraction occurs in different, circular transmission zones inside the colony, responsible for the presence of intensity rings in both the numerically reconstructed intensity pattern and the experimentally recorded Fresnel diffraction pattern. The central intensity ring occurs on the boundaries of the bacterial colony center, where the oldest bacterial cells are located and the highest concentration of extracellular substance is found. The second intensity ring results from the light diffraction on the bacterial colony edge. Moreover, the presence of additional radial spokes between the two intensity rings in both patterns results from the internal heterogeneity of the colony. This heterogeneity is caused by the migration of young cells from the colony center, leading to local changes in refractive index.

The results demonstrate that the reconstructed phase and intensity patterns of the light diffracted by bacterial colonies exhibit similar unique features as the Fresnel diffraction patterns, enabling identification of bacteria with nearly 99% accuracy. Therefore, they can also be used for bacterial identification. However, for experimentally recorded Fresnel patterns, an additional speckle effect is observed. It is probably caused by the roughness of the colony surface [30]. Moreover, the digital holograms of bacterial colonies were recorded using a light source with a wavelength of 405 nm while the Fresnel diffraction patterns were recorded at a wavelength of 635 nm. This contributes to the differences between the reconstructed diffraction patterns and these recorded in previously proposed optical system. Changes in the wavelength of the illuminating beam affect the diffraction patterns [[31]]. Long wavelengths lead to wider and sparser patterns, while the overall diffraction pattern size is decreasing. These observations are also confirmed by our results. Moreover, in our previous optical systems a converging illuminating beam was used, while in PSDIH, the illuminating beam was transformed by a pinhole and diverging. This difference can also be responsible for the discrepancy in the lateral size of diffraction patterns obtained by these two techniques. However, it should be pointed out that, although there are some differences between the reconstructed and recorded Fresnel patterns, both patterns exhibit similar species-dependent features.

### The PCA analysis of the analyzed optical signatures of bacterial colonies

**Fig 8:**
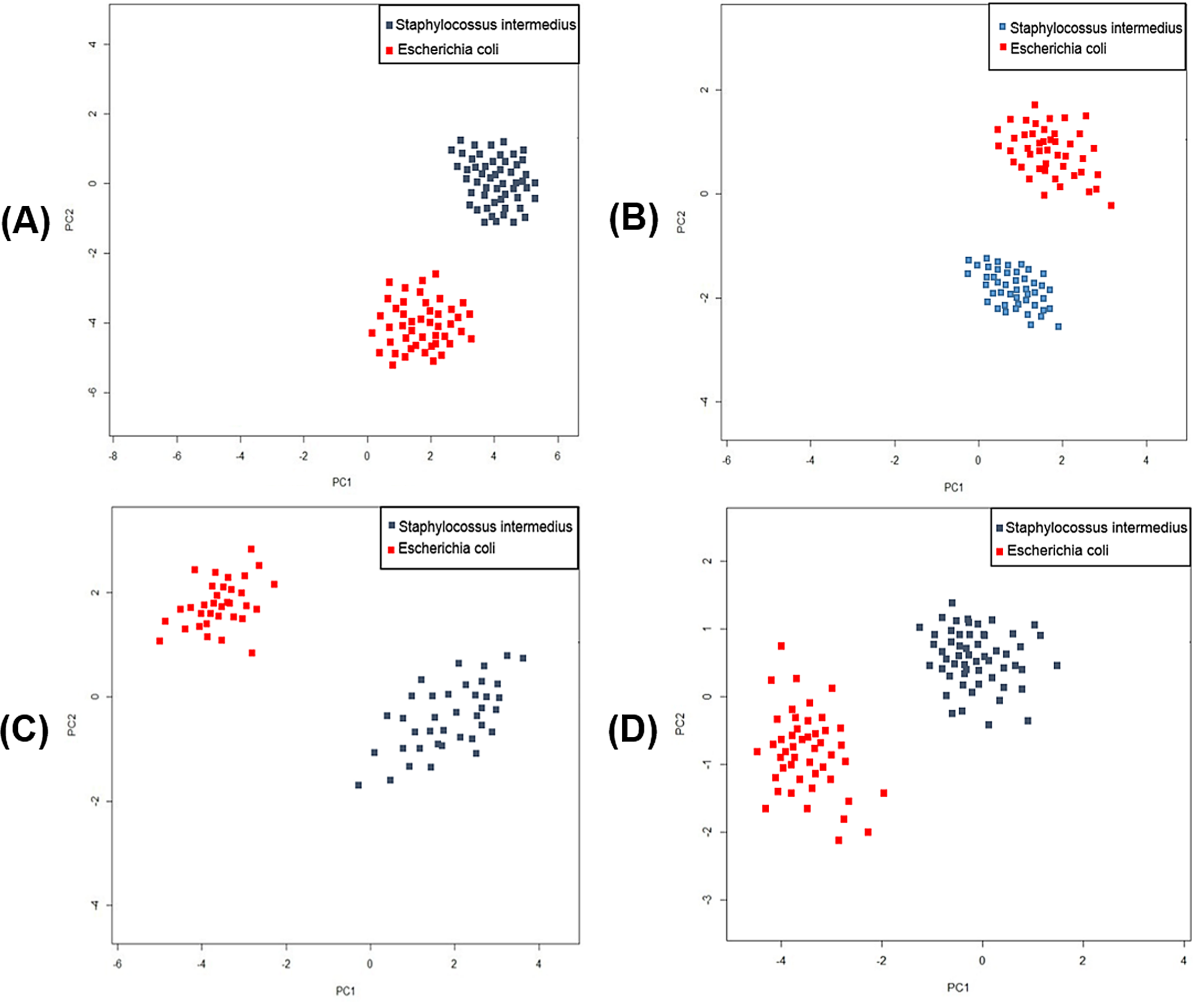
**Representative results of PCA analysis of the optical signatures**: recorded digital holograms (A), amplitude (B) and phase (C) images, reconstructed diffraction intensity patterns (D) of both analyzed bacteria species colonies.

In case of PSDIH holograms of bacterial colonies, the area occupied by the colony was limited by partitioning zones, and the features were extracted according to the algorithm described in previous Sections. The first and second principal components of the holograms are plotted and two different analyzed bacteria species have been marked by different colors (see Fig 8A). Obtained results on the separation of data points, have shown that extracted features of the holograms are differentiating examined bacteria species. Moreover, the concentration of the data points for both bacteria species suggests the repeatability of recorded PSDIH holograms for different bacterial colonies of the same species.

The same tendency (see Fig 8B) is observed in case of the PCA analysis of the reconstructed amplitude images of bacterial colonies. The separation of data points is affected directly by significantly different transmission properties of bacterial colonies. Moreover, the high concentration of the data points for *S*. *intermedius* can be associated with low transmittance observed for all colonies of these bacteria species.

In case of the reconstructed phase images of bacterial colonies (see Fig 8C), the analysis of first and second principal components has shown the separation of the data points for two bacteria species. So, the species differentiation based on reconstructed optical signatures, is possible. In this case the concentration of the data points is lower, than in case of amplitude images of bacterial colonies. It can be associated with the differences of the phase distribution among each bacterial colony, caused by local changes of the colony refractive index affected by random bacterial cells orientation, extracellular material spatial distribution and colony profile changes during the colony evolution.

The reconstructed diffraction intensity patterns of both bacteria species at chosen distance 2 cm from the bacterial colony were also analyzed in term of PCA (see Fig 8D). The obtained results, as in case of previous analyzed optical signatures, have shown that it is possible to differentiate the bacteria species basing on features extracted from reconstructed diffraction patterns.

Performed analysis of the features extracted from the optical signatures of bacterial colonies obtained by PSDIH points out the possibility of differentiation of two analyzed bacteria species.

## Conclusions

The main goal of this work was to demonstrate the novel application of point-source digital in-line holography for the characterization of bacterial colonies and its potential use for microbiological investigation. The main advantage of digital holography is the possibility to reconstruct the amplitude and phase properties of examined objects, as well as the amplitude and phase patterns of the optical field scattered/diffracted by the object in a chosen observation plane behind the object from one single digital hologram. The proposed method non-contact and non-destructive, offers label-free examination of bacterial samples. To the best of our knowledge, this report is the first attempt at characterizing the species-dependent properties of bacterial colonies and their diffraction patterns by digital in-line holography.

The analysis of the amplitude and phase patterns inside the colony revealed their unique species-dependent optical properties, which are associated with the internal structure and geometry of the bacterial colony. Moreover, the single measurement digital hologram recording and its numerical reconstruction enable obtaining a reference database of the bacterial diffraction signatures from all observation spaces. As such, it allows for the extraction of additional differentiating features, in contrast to the already proposed methods based on single scattering/diffraction patterns recorded in a fixed observation plane. This method can provide new optical discriminators for bacterial species, which can extend the classification vector and improve the bacterial identification accuracy. Comparison of the reconstructed diffraction intensity patterns obtained with PSDIHM with previously recorded Fresnel diffraction patterns of the same colonies demonstrated that both patterns are highly similar and highlight the same unique features associated with each bacterial species. This indicates the possibility of using digital holography for bacterial identification. The potential of bacteria species differentiation by digital holographic signatures was also demonstrated by means of the PCA analysis of examined optical signatures of bacterial colonies. It was shown that the analyzed optical signatures obtained by digital holography exhibit unique species-dependent features.

Decreasing the incubation time can improve the transmission properties of the colonies and the quality of the numerically reconstructed amplitude and phase patterns. Future research will be focused on more extended investigation of potential application of digital holographic sensors for different bacteria species/strains characterization. Proposed method has non-contact and non-destructive character, and enables label-free investigation of bacteria samples.

## References

[1] Ivnitski D, Abdel-Hamid I, Atanasov P, Wilkins E. Biosensors for detection of pathogenic bacteria. Biosens Bioelectron. 1999;14: 599–624. 10.1016/S0956-5663(99)00039-1 10230031

[2] Lazcka O, Del Campo FJ, Muñoz FX. Pathogen detection: a perspective of traditional methods and biosensors. Biosens Bioelectron. 2007;22: 1205–17. 10.1016/j.bios.2006.06.036 16934970

[3] Kok J, Thomas LC, Olma T, Chen SCA, Iredell JR. Identification of bacteria in blood culture broths using matrix-assisted laser desorption-ionization Sepsityper^TM^ and time of flight mass spectrometry. PLoS One. Public Library of Science; 2011;6: e23285 10.1371/journal.pone.0023285 PMC3156714 21858058

[4] Dartnell LR, Roberts TA, Moore G, Ward JM, Muller J-P. Fluorescence characterization of clinically-important bacteria. PLoS One. Public Library of Science; 2013;8: e75270 10.1371/journal.pone.0075270 24098687 PMC3787103

[5] Banada PP, Guo S, Bayraktar B, Bae E, Rajwa B, Robinson JP, et al Optical forward-scattering for detection of Listeria monocytogenes and other Listeria species. Biosens Bioelectron. 2007;22: 1664–71. 10.1016/j.bios.2006.07.028 16949268

[6] Banada PP, Huff K, Bae E, Rajwa B, Aroonnual A, Bayraktar B, et al Label-free detection of multiple bacterial pathogens using light-scattering sensor. Biosens Bioelectron. 2009;24: 1685–92. 10.1016/j.bios.2008.08.053 18945607

[7] Buzalewicz I, Podbielska H. Optical Sensing of Bacteria by Means of Light Diffraction Frontiers in Optics 2010/Laser Science XXVI. Washington, D.C.: OSA; 2010 p. JWA13. 10.1364/FIO.2010.JWA13

[8] Buzalewicz I, Wieliczko A, Podbielska H. Influence of various growth conditions on Fresnel diffraction patterns of bacteria colonies examined in the optical system with converging spherical wave illumination. Opt Express. Optical Society of America; 2011;19: 21768–21785. 10.1364/OE.19.021768 22109028

[9] Marcoux PR, Dupoy M, Cuer A, Kodja JL, Lefebvre A, Licari F, et al Optical forward-scattering for identification of bacteria within microcolonies. Appl Microbiol Biotechnol. Springer-Verlag, New York, USA; 2014;98: 2243–2254. 10.1007/s00253-013-5495-4 24413976

[10] Tang Y, Kim H, Singh AK, Aroonnual A, Bae E, Rajwa B, et al Light scattering sensor for direct identification of colonies of Escherichia coli serogroups O26, O45, O103, O111, O121, O145 and O157. PLoS One. Public Library of Science; 2014;9: e105272 10.1371/journal.pone.0105272 25136836 PMC4138183

[11] Buzalewicz I, Wieliczko A, Bednarek KJ, Podbielska H. Diffraction signature of bacteria colonies and the influence of different incubation conditions Frontiers in Optics 2011/Laser Science XXVII. Washington, D.C.: OSA; 2011 p. JWA6. 10.1364/FIO.2011.JWA6

[12] Podbielska H, Buzalewicz I, Suchwalko A, Wieliczko A. Bacteria Classification by Means of the Statistical Analysis of Fresnel Diffraction Patterns of Bacteria Colonies Biomedical Optics and 3-D Imaging. Washington, D.C.: OSA; 2012. p. BSu5A.5. 10.1364/BIOMED.2012.BSu5A.5

[13] Suchwalko A, Buzalewicz I, Podbielska H. Computer-based classification of bacteria species by analysis of their colonies Fresnel diffraction patterns. 2012;8212: 82120R–82120R–13. 10.1117/12.907420

[14] Suchwalko A, Buzalewicz I, Podbielska H. Statistical identification of bacteria species In: Méndez-Vilas A, editor. Microbial pathogens and strategies for combating them: science, technology and education. Badajoz, Spain: Formatex Research Center; 2013 pp. 711–721.

[15] Suchwalko A, Buzalewicz I, Podbielska H. Identification of bacteria species by using morphological and textural properties of bacterial colonies diffraction patterns. In: Remondino F, Shortis MR, Beyerer J, Puente León F, editors. Proceedings of SPIE. 2013. pp. 87911M–1–87911M–7. 10.1117/12.2020337

[16] Suchwalko A, Buzalewicz I, Wieliczko A, Podbielska H. Bacteria species identification by the statistical analysis of bacterial colonies Fresnel patterns. Opt Express. 2013;21: 11322 10.1364/OE.21.011322 23669989

[17] Suchwałko A, Buzalewicz I, Podbielska H. Bacteria identification in an optical system with optimized diffraction pattern registration condition supported by enhanced statistical analysis. Opt Express. 2014;22: 26312 10.1364/OE.22.026312 25401664

[18] Minoni U, Signoroni A, Nassini G. On the application of optical forward-scattering to bacterial identification in an automated clinical analysis perspective. Biosens Bioelectron. 2015;68: 536–43. 10.1016/j.bios.2015.01.047 25643595

[19] Kim MK. Digital Holographic Microscopy. Imaging Microsc. 2011;162: 129–147. 10.1007/978-1-4419-7793-9

[20] Yaroslavsky L. Digital Holography and Digital Image Processing: Principles, Methods, Algorithms. 1st ed. Springer US; 2003 10.1007/978-1-4757-4988-5

[21] Qu W, Choo Chee O, Yu Y, Asundi A. Recording and reconstruction of digital Gabor hologram. Opt—Int J Light Electron Opt. 2010;121: 2179–2184. 10.1016/j.ijleo.2009.11.004

[22] Garcia-Sucerquia J, Xu W, Jericho SK, Klages P, Jericho MH, Kreuzer HJ. Digital in-line holographic microscopy. Appl Opt. Optical Society of America; 2006;45: 836 10.1364/AO.45.000836 16512525

[23] Xu W, Jericho MH, Meinertzhagen IA, Kreuzer HJ. Digital in-line holography for biological applications. Proc Natl Acad Sci U S A. 2001;98: 11301–5. 10.1073/pnas.191361398 11572982 PMC58724

[24] Jericho MH, Kreuzer HJ, Kanka M, Riesenberg R. Quantitative phase and refractive index measurements with point-source digital in-line holographic microscopy. Appl Opt. Optical Society of America; 2012;51: 1503–15. 10.1364/AO.51.001503 22505068

[25] Manfred H. Jericho HJK. Coherent Light Microscopy Ferraro P, Wax A, Zalevsky Z, editors. Berlin, Heidelberg: Springer Berlin Heidelberg; 2011 10.1007/978-3-642-15813-1

[26] Kanka M, Riesenberg R, Kreuzer HJ. Reconstruction of high-resolution holographic microscopic images. Opt Lett. Optical Society of America; 2009;34: 1162 10.1364/OL.34.001162 19370104

[27] Goodman JW. Introduction to Fourier Optics. Roberts & Company; 2005.

[28] ImageJ [Internet]. [cited 24 Nov 2015]. Available: http://imagej.nih.gov/ij/

[29] R: The R Project for Statistical Computing [Internet]. [cited 24 Nov 2015]. Available: https://www.r-project.org/

[30] Buzalewicz I, Liżewski K, Kujawińska M, Podbielska H. Degeneration of Fraunhofer diffraction on bacterial colonies due to their light focusing properties examined in the digital holographic microscope system. Opt Express. OSA; 2013;21: 26493 10.1364/OE.21.026493 24216870

[31] Kim H, Doh I-J, Bhunia AK, King GB, Bae E. Scalar diffraction modeling of multispectral forward scatter patterns from bacterial colonies. Opt Express. Optical Society of America; 2015;23: 8545–54. 10.1364/OE.23.008545 25968692

